# 9,9-Bis[4-(prop-2-yn­yloxy)phen­yl]-9*H*-fluorene

**DOI:** 10.1107/S1600536810025833

**Published:** 2010-07-07

**Authors:** Kiramat Shah, Muhammad Raza Shah, Seik Weng Ng

**Affiliations:** a H. E. J. Research Institute of Chemistry, International Center for Chemical and Biological Sciences, University of Karachi, Karachi 75270, Pakistan; b Department of Chemistry, University of Malaya, 50603 Kuala Lumpur, Malaysia

## Abstract

In the title compound, C_31_H_22_O_2_, the bond angle at the C atom belonging to the five-membered ring of the fluorene system is opened to 112.64 (12)°. The two benzene rings are twisted with respect to the fluorene ring system at dihedral angles of 72.81 (6) and 81.83 (6)°. One C_ar­yl_—O—C—C  fragment is extended, with a C—O—C—C torsion angle of −178.77 (13)°, but the other C_ar­yl_—O—C—C  fragment is bent, with a C—O—C—C torsion angle of 64.78 (19)°. Inter­molecular weak C—H⋯O hydrogen bonding is present in the crystal structure.

## Related literature

For the synthesis of copolyethers having 1,3,4-oxadiazole rings and fluorene groups from the polymerization of 9,9-bis­(4-propargyloxyphen­yl)fluorene, see: Hamciuc *et al.* (2009 ▶). For a related structure, see: Shah *et al.* (2010 ▶).
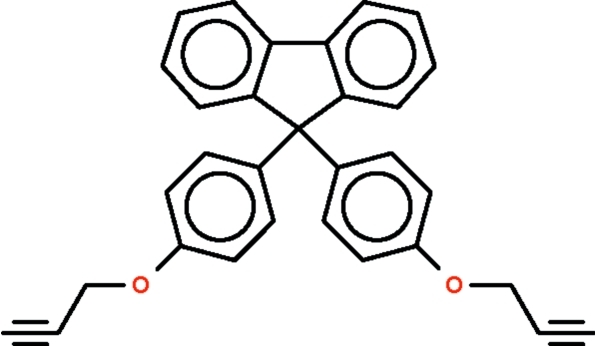


## Experimental

### 

#### Crystal data

C_31_H_22_O_2_
*M* *_r_* = 426.49
Orthorhombic, 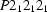

*a* = 11.0047 (8) Å
*b* = 12.9686 (9) Å
*c* = 15.4371 (11) Å
*V* = 2203.1 (3) Å^3^
*Z* = 4
Mo *K*α radiation
μ = 0.08 mm^−1^
*T* = 100 K
0.35 × 0.25 × 0.15 mm

#### Data collection

Bruker SMART APEX diffractometer
21245 measured reflections
2861 independent reflections
2658 reflections with *I* > 2σ(*I*)
*R*_int_ = 0.042

#### Refinement

*R*[*F* ^2^ > 2σ(*F* ^2^)] = 0.032
*wR*(*F* ^2^) = 0.082
*S* = 1.04
2861 reflections
306 parameters
2 restraints
H atoms treated by a mixture of independent and constrained refinement
Δρ_max_ = 0.18 e Å^−3^
Δρ_min_ = −0.20 e Å^−3^
Absolute structure: 2204 Friedel pairs were merged
Flack parameter: ?
Rogers parameter: ?

### 

Data collection: *APEX2* (Bruker, 2009 ▶); cell refinement: *SAINT* (Bruker, 2009 ▶); data reduction: *SAINT*; program(s) used to solve structure: *SHELXS97* (Sheldrick, 2008 ▶); program(s) used to refine structure: *SHELXL97* (Sheldrick, 2008 ▶); molecular graphics: *X-SEED* (Barbour, 2001 ▶); software used to prepare material for publication: *publCIF* (Westrip, 2010 ▶).

## Supplementary Material

Crystal structure: contains datablocks global, I. DOI: 10.1107/S1600536810025833/xu2792sup1.cif

Structure factors: contains datablocks I. DOI: 10.1107/S1600536810025833/xu2792Isup2.hkl

Additional supplementary materials:  crystallographic information; 3D view; checkCIF report

## Figures and Tables

**Table 1:** Hydrogen-bond geometry (Å, °)

*D*—H⋯*A*	*D*—H	H⋯*A*	*D*⋯*A*	*D*—H⋯*A*
C29—H29*A*⋯O1^i^	0.99	2.46	3.358 (2)	151

Symmetry code: (i) 
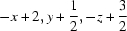
.

**Fig. 1.**
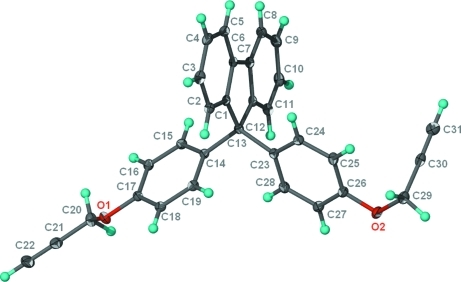
Thermal ellipsoid plot (Barbour, 2001) of C31H22O2 at the 50% probability level; hydrogen atoms are drawn as spheres of arbitrary radius.

## supplementary crystallographic information

### Comment

9,9-Bis(4-propargyloxyphenyl)fluorene is the mononer required for the synthesis of other copolyethers (Hamciuc *et al.*, 2009); these polymers typically possess good solubility in inorganic solvents, high thermal stability and high glass transition temperatures. The compound is readily synthesized from commercially available 9,9-bis(4-hydroxyphenyl)fluorene and propargyl bromide. A previous report detailed the structure of the product of its reaction with *t*-butyl bromoacetate (Shah *et al.*, 2010). In the C_31_H_22_O_2_ molecule (Scheme I, Fig. 1), the angle at the carbon atom belonging to the five-membered fluorenyl ring that is connected to two *p*-phenylene rings is open to 116.1 (1) ° by the rings. One four-atom C_aryl_–O–C–C≡ fragment is coplanar with the ring [C—O–C–C torsion angle 1.2 (1) °] but the other is bent [torsion angle 64.8 (2) °].

### Experimental

9,9-Bis(4-hydroxyphenyl)fluorene (0.5 g, 1.4 mmol) was dissolved in acetone (25 ml) to give a clear solution. Potassium carbonate (0.7 g, 5 mmol) was added and the mixture stirred for an hour. Propargyl bromide (0.7 ml, 5 mmol) was added and stirring continued overnight. The mixture was filtered; prismatic crystals separated from the solution in 80% yield.

### Refinement

The acetylenic H-atoms were located in a difference Fourier map, and were refined with a distance restraint of C–H 0.95±0.01 Å; their temperature factors were refined. Other H atoms were placed in calculated positions [C–H 0.95–0.99 Å, *U*(H) = 1.2*U*(C)] and were included in the refinement in the riding model approximation. Friedel pairs were merged as no significant anomalous scattering.

### Crystal data

**Table:**

C_31_H_22_O_2_	*F*(000) = 896
*M**_r_* = 426.49	*D*_x_ = 1.286 Mg m^−^^3^
Orthorhombic, *P*2_1_2_1_2_1_	Mo *K*α radiation, λ = 0.71073 Å
Hall symbol: P 2ac 2ab	Cell parameters from 7024 reflections
*a* = 11.0047 (8) Å	θ = 2.3–27.7°
*b* = 12.9686 (9) Å	µ = 0.08 mm^−^^1^
*c* = 15.4371 (11) Å	*T* = 100 K
*V* = 2203.1 (3) Å^3^	Block, colorless
*Z* = 4	0.35 × 0.25 × 0.15 mm

### Data collection

**Table:**

Bruker SMART APEX diffractometer	2658 reflections with *I* > 2σ(*I*)
Radiation source: fine-focus sealed tube	*R*_int_ = 0.042
graphite	θ_max_ = 27.5°, θ_min_ = 2.1°
ω scans	*h* = −14→14
21245 measured reflections	*k* = −16→16
2861 independent reflections	*l* = −19→20

### Refinement

**Table:**

Refinement on *F*^2^	Secondary atom site location: difference Fourier map
Least-squares matrix: full	Hydrogen site location: inferred from neighbouring sites
*R*[*F*^2^ > 2σ(*F*^2^)] = 0.032	H atoms treated by a mixture of independent and constrained refinement
*wR*(*F*^2^) = 0.082	*w* = 1/[σ^2^(*F*_o_^2^) + (0.0504*P*)^2^ + 0.3105*P*] where *P* = (*F*_o_^2^ + 2*F*_c_^2^)/3
*S* = 1.04	(Δ/σ)_max_ = 0.001
2861 reflections	Δρ_max_ = 0.18 e Å^−^^3^
306 parameters	Δρ_min_ = −0.20 e Å^−^^3^
2 restraints	Absolute structure: 2204 Friedel pairs were merged
Primary atom site location: structure-invariant direct methods	

### Fractional atomic coordinates and isotropic or equivalent isotropic displacement parameters (Å^2^)

**Table:**

	*x*	*y*	*z*	*U*_iso_*/*U*_eq_	
O1	1.24715 (9)	0.37529 (9)	1.06380 (7)	0.0184 (3)	
O2	0.72277 (11)	0.74489 (10)	0.66169 (7)	0.0228 (3)	
C1	0.84334 (14)	0.71897 (12)	1.07308 (10)	0.0146 (3)	
C2	0.93879 (15)	0.78830 (12)	1.08005 (11)	0.0174 (3)	
H2	1.0072	0.7832	1.0428	0.021*	
C3	0.93255 (15)	0.86561 (13)	1.14272 (11)	0.0200 (3)	
H3	0.9979	0.9130	1.1488	0.024*	
C4	0.83115 (16)	0.87398 (13)	1.19668 (11)	0.0205 (3)	
H4	0.8278	0.9276	1.2385	0.025*	
C5	0.73513 (16)	0.80494 (13)	1.18992 (11)	0.0188 (3)	
H5	0.6660	0.8111	1.2264	0.023*	
C6	0.74238 (14)	0.72624 (13)	1.12841 (10)	0.0156 (3)	
C7	0.65875 (14)	0.64169 (13)	1.10835 (10)	0.0169 (3)	
C8	0.54672 (15)	0.61615 (14)	1.14465 (11)	0.0206 (3)	
H8	0.5128	0.6565	1.1900	0.025*	
C9	0.48567 (15)	0.52999 (15)	1.11285 (12)	0.0226 (4)	
H9	0.4097	0.5109	1.1372	0.027*	
C10	0.53482 (15)	0.47162 (14)	1.04579 (12)	0.0211 (4)	
H10	0.4922	0.4129	1.0251	0.025*	
C11	0.64577 (15)	0.49837 (13)	1.00872 (11)	0.0189 (3)	
H11	0.6782	0.4593	0.9621	0.023*	
C12	0.70800 (14)	0.58268 (13)	1.04078 (10)	0.0157 (3)	
C13	0.82912 (13)	0.62794 (12)	1.01042 (10)	0.0143 (3)	
C14	0.93668 (14)	0.55407 (12)	1.02154 (10)	0.0145 (3)	
C15	0.93030 (15)	0.46837 (13)	1.07548 (11)	0.0167 (3)	
H15	0.8553	0.4519	1.1027	0.020*	
C16	1.03105 (14)	0.40609 (13)	1.09059 (10)	0.0170 (3)	
H16	1.0248	0.3477	1.1275	0.020*	
C17	1.14088 (14)	0.43008 (13)	1.05113 (11)	0.0151 (3)	
C18	1.14950 (14)	0.51457 (12)	0.99593 (11)	0.0160 (3)	
H18	1.2243	0.5303	0.9682	0.019*	
C19	1.04803 (14)	0.57562 (12)	0.98172 (10)	0.0160 (3)	
H19	1.0542	0.6334	0.9441	0.019*	
C20	1.23372 (16)	0.26883 (13)	1.08654 (12)	0.0210 (4)	
H20A	1.1907	0.2313	1.0399	0.025*	
H20B	1.1859	0.2623	1.1406	0.025*	
C21	1.35523 (17)	0.22575 (14)	1.09882 (11)	0.0222 (4)	
C22	1.4531 (2)	0.19125 (17)	1.11001 (14)	0.0342 (5)	
C23	0.81257 (14)	0.66357 (13)	0.91600 (10)	0.0153 (3)	
C24	0.76842 (14)	0.76168 (13)	0.89833 (10)	0.0163 (3)	
H24	0.7588	0.8092	0.9447	0.020*	
C25	0.73786 (15)	0.79255 (13)	0.81480 (11)	0.0186 (3)	
H25	0.7075	0.8600	0.8043	0.022*	
C26	0.75237 (15)	0.72339 (14)	0.74710 (10)	0.0182 (3)	
C27	0.79946 (15)	0.62612 (15)	0.76239 (11)	0.0210 (4)	
H27	0.8119	0.5799	0.7155	0.025*	
C28	0.82856 (15)	0.59607 (13)	0.84623 (11)	0.0192 (3)	
H28	0.8597	0.5288	0.8563	0.023*	
C29	0.67080 (16)	0.84363 (14)	0.64399 (11)	0.0221 (4)	
H29A	0.6628	0.8518	0.5805	0.027*	
H29B	0.7272	0.8977	0.6649	0.027*	
C30	0.55120 (16)	0.86010 (14)	0.68398 (11)	0.0203 (3)	
C31	0.45729 (17)	0.87683 (15)	0.71807 (12)	0.0254 (4)	
H22	1.5337 (13)	0.167 (2)	1.1183 (19)	0.073 (10)*	
H31	0.3818 (13)	0.8907 (17)	0.7453 (13)	0.036 (6)*	

### Atomic displacement parameters (Å^2^)

**Table:**

	*U*^11^	*U*^22^	*U*^33^	*U*^12^	*U*^13^	*U*^23^
O1	0.0141 (5)	0.0153 (6)	0.0257 (6)	0.0011 (5)	0.0006 (5)	0.0027 (5)
O2	0.0289 (6)	0.0249 (6)	0.0146 (5)	0.0056 (5)	−0.0034 (5)	0.0000 (5)
C1	0.0168 (7)	0.0140 (7)	0.0130 (7)	0.0024 (6)	−0.0032 (6)	0.0020 (6)
C2	0.0182 (7)	0.0176 (8)	0.0164 (8)	−0.0001 (6)	−0.0016 (6)	0.0003 (6)
C3	0.0233 (8)	0.0157 (8)	0.0209 (8)	−0.0031 (7)	−0.0052 (7)	0.0008 (7)
C4	0.0292 (8)	0.0168 (8)	0.0154 (7)	0.0034 (7)	−0.0040 (7)	−0.0016 (6)
C5	0.0212 (8)	0.0205 (8)	0.0147 (7)	0.0045 (7)	−0.0007 (6)	0.0001 (7)
C6	0.0161 (7)	0.0167 (8)	0.0140 (7)	0.0031 (6)	−0.0021 (6)	0.0020 (6)
C7	0.0165 (7)	0.0184 (8)	0.0159 (7)	0.0021 (6)	−0.0024 (6)	0.0027 (6)
C8	0.0160 (7)	0.0267 (9)	0.0192 (8)	0.0023 (7)	−0.0003 (6)	0.0019 (7)
C9	0.0140 (7)	0.0283 (9)	0.0256 (9)	−0.0019 (7)	0.0001 (7)	0.0063 (8)
C10	0.0167 (7)	0.0196 (8)	0.0269 (9)	−0.0035 (6)	−0.0053 (7)	0.0031 (7)
C11	0.0171 (7)	0.0181 (8)	0.0215 (8)	0.0007 (6)	−0.0025 (7)	0.0006 (7)
C12	0.0145 (7)	0.0161 (8)	0.0164 (7)	0.0017 (6)	−0.0026 (6)	0.0036 (6)
C13	0.0144 (7)	0.0131 (7)	0.0153 (7)	0.0007 (6)	−0.0004 (6)	0.0003 (6)
C14	0.0146 (7)	0.0151 (8)	0.0138 (7)	0.0007 (6)	−0.0012 (6)	−0.0017 (6)
C15	0.0154 (7)	0.0188 (8)	0.0158 (8)	−0.0021 (6)	0.0008 (6)	0.0007 (6)
C16	0.0186 (7)	0.0163 (8)	0.0159 (8)	−0.0001 (6)	−0.0007 (6)	0.0031 (6)
C17	0.0149 (7)	0.0142 (8)	0.0162 (7)	0.0000 (6)	−0.0018 (6)	−0.0032 (6)
C18	0.0154 (7)	0.0160 (8)	0.0166 (8)	−0.0025 (6)	0.0014 (6)	−0.0009 (6)
C19	0.0192 (7)	0.0126 (7)	0.0160 (8)	−0.0018 (6)	0.0005 (6)	0.0012 (6)
C20	0.0219 (8)	0.0149 (8)	0.0263 (9)	−0.0003 (7)	−0.0036 (7)	0.0033 (7)
C21	0.0292 (9)	0.0181 (8)	0.0195 (8)	0.0046 (7)	0.0028 (7)	0.0010 (7)
C22	0.0337 (10)	0.0411 (12)	0.0279 (10)	0.0200 (10)	0.0063 (9)	0.0081 (9)
C23	0.0137 (7)	0.0172 (8)	0.0151 (7)	−0.0003 (6)	−0.0010 (6)	−0.0002 (6)
C24	0.0173 (7)	0.0151 (8)	0.0165 (7)	0.0005 (6)	−0.0011 (6)	−0.0031 (6)
C25	0.0203 (8)	0.0151 (8)	0.0205 (8)	0.0011 (7)	−0.0041 (7)	0.0005 (7)
C26	0.0172 (7)	0.0227 (8)	0.0149 (7)	−0.0005 (6)	−0.0009 (6)	−0.0002 (7)
C27	0.0224 (8)	0.0235 (9)	0.0169 (8)	0.0036 (7)	−0.0017 (6)	−0.0040 (7)
C28	0.0208 (8)	0.0161 (8)	0.0206 (8)	0.0037 (6)	−0.0023 (7)	−0.0021 (7)
C29	0.0247 (8)	0.0233 (9)	0.0184 (8)	0.0012 (7)	−0.0012 (7)	0.0044 (7)
C30	0.0265 (8)	0.0175 (8)	0.0170 (8)	−0.0020 (7)	−0.0052 (7)	0.0025 (7)
C31	0.0265 (9)	0.0253 (10)	0.0243 (9)	−0.0038 (8)	0.0013 (7)	−0.0011 (8)

### Geometric parameters (Å, °)

**Table:**

O1—C17	1.3824 (18)	C14—C19	1.399 (2)
O1—C20	1.432 (2)	C15—C16	1.391 (2)
O2—C26	1.387 (2)	C15—H15	0.9500
O2—C29	1.429 (2)	C16—C17	1.389 (2)
C1—C2	1.387 (2)	C16—H16	0.9500
C1—C6	1.405 (2)	C17—C18	1.391 (2)
C1—C13	1.534 (2)	C18—C19	1.386 (2)
C2—C3	1.395 (2)	C18—H18	0.9500
C2—H2	0.9500	C19—H19	0.9500
C3—C4	1.397 (2)	C20—C21	1.462 (2)
C3—H3	0.9500	C20—H20A	0.9900
C4—C5	1.389 (2)	C20—H20B	0.9900
C4—H4	0.9500	C21—C22	1.179 (3)
C5—C6	1.396 (2)	C22—H22	0.949 (10)
C5—H5	0.9500	C23—C24	1.389 (2)
C6—C7	1.465 (2)	C23—C28	1.399 (2)
C7—C8	1.394 (2)	C24—C25	1.391 (2)
C7—C12	1.403 (2)	C24—H24	0.9500
C8—C9	1.393 (3)	C25—C26	1.386 (2)
C8—H8	0.9500	C25—H25	0.9500
C9—C10	1.392 (3)	C26—C27	1.384 (2)
C9—H9	0.9500	C27—C28	1.389 (2)
C10—C11	1.392 (2)	C27—H27	0.9500
C10—H10	0.9500	C28—H28	0.9500
C11—C12	1.382 (2)	C29—C30	1.469 (2)
C11—H11	0.9500	C29—H29A	0.9900
C12—C13	1.530 (2)	C29—H29B	0.9900
C13—C14	1.532 (2)	C30—C31	1.180 (3)
C13—C23	1.540 (2)	C31—H31	0.949 (10)
C14—C15	1.390 (2)		
			
C17—O1—C20	116.28 (12)	C14—C15—H15	119.2
C26—O2—C29	117.13 (13)	C16—C15—H15	119.2
C2—C1—C6	120.56 (15)	C17—C16—C15	119.34 (15)
C2—C1—C13	128.68 (14)	C17—C16—H16	120.3
C6—C1—C13	110.75 (13)	C15—C16—H16	120.3
C1—C2—C3	118.85 (15)	O1—C17—C16	124.00 (14)
C1—C2—H2	120.6	O1—C17—C18	115.70 (14)
C3—C2—H2	120.6	C16—C17—C18	120.29 (15)
C2—C3—C4	120.58 (16)	C19—C18—C17	119.46 (15)
C2—C3—H3	119.7	C19—C18—H18	120.3
C4—C3—H3	119.7	C17—C18—H18	120.3
C5—C4—C3	120.86 (15)	C18—C19—C14	121.46 (15)
C5—C4—H4	119.6	C18—C19—H19	119.3
C3—C4—H4	119.6	C14—C19—H19	119.3
C4—C5—C6	118.61 (15)	O1—C20—C21	107.81 (14)
C4—C5—H5	120.7	O1—C20—H20A	110.1
C6—C5—H5	120.7	C21—C20—H20A	110.1
C5—C6—C1	120.52 (15)	O1—C20—H20B	110.1
C5—C6—C7	130.92 (15)	C21—C20—H20B	110.1
C1—C6—C7	108.56 (14)	H20A—C20—H20B	108.5
C8—C7—C12	120.73 (16)	C22—C21—C20	179.0 (2)
C8—C7—C6	130.43 (16)	C21—C22—H22	177 (2)
C12—C7—C6	108.84 (14)	C24—C23—C28	117.77 (15)
C9—C8—C7	118.38 (17)	C24—C23—C13	120.14 (14)
C9—C8—H8	120.8	C28—C23—C13	121.74 (14)
C7—C8—H8	120.8	C23—C24—C25	122.01 (15)
C8—C9—C10	120.73 (16)	C23—C24—H24	119.0
C8—C9—H9	119.6	C25—C24—H24	119.0
C10—C9—H9	119.6	C26—C25—C24	118.99 (15)
C11—C10—C9	120.73 (17)	C26—C25—H25	120.5
C11—C10—H10	119.6	C24—C25—H25	120.5
C9—C10—H10	119.6	C27—C26—O2	115.68 (15)
C12—C11—C10	118.97 (16)	C27—C26—C25	120.28 (15)
C12—C11—H11	120.5	O2—C26—C25	124.04 (15)
C10—C11—H11	120.5	C26—C27—C28	120.06 (16)
C11—C12—C7	120.44 (15)	C26—C27—H27	120.0
C11—C12—C13	128.72 (15)	C28—C27—H27	120.0
C7—C12—C13	110.80 (14)	C27—C28—C23	120.85 (16)
C12—C13—C14	113.50 (13)	C27—C28—H28	119.6
C12—C13—C1	101.01 (12)	C23—C28—H28	119.6
C14—C13—C1	109.36 (12)	O2—C29—C30	114.11 (14)
C12—C13—C23	107.59 (12)	O2—C29—H29A	108.7
C14—C13—C23	112.64 (12)	C30—C29—H29A	108.7
C1—C13—C23	112.21 (13)	O2—C29—H29B	108.7
C15—C14—C19	117.84 (14)	C30—C29—H29B	108.7
C15—C14—C13	121.86 (14)	H29A—C29—H29B	107.6
C19—C14—C13	120.16 (14)	C31—C30—C29	177.1 (2)
C14—C15—C16	121.61 (15)	C30—C31—H31	179.6 (16)
			
C6—C1—C2—C3	−0.3 (2)	C12—C13—C14—C15	−17.1 (2)
C13—C1—C2—C3	−179.70 (15)	C1—C13—C14—C15	94.84 (17)
C1—C2—C3—C4	−0.9 (2)	C23—C13—C14—C15	−139.66 (15)
C2—C3—C4—C5	0.8 (3)	C12—C13—C14—C19	167.42 (14)
C3—C4—C5—C6	0.5 (2)	C1—C13—C14—C19	−80.65 (17)
C4—C5—C6—C1	−1.7 (2)	C23—C13—C14—C19	44.9 (2)
C4—C5—C6—C7	177.91 (16)	C19—C14—C15—C16	0.7 (2)
C2—C1—C6—C5	1.6 (2)	C13—C14—C15—C16	−174.94 (15)
C13—C1—C6—C5	−178.88 (14)	C14—C15—C16—C17	0.2 (3)
C2—C1—C6—C7	−178.09 (14)	C20—O1—C17—C16	27.2 (2)
C13—C1—C6—C7	1.44 (17)	C20—O1—C17—C18	−153.71 (14)
C5—C6—C7—C8	0.7 (3)	C15—C16—C17—O1	177.96 (15)
C1—C6—C7—C8	−179.64 (16)	C15—C16—C17—C18	−1.1 (2)
C5—C6—C7—C12	−179.73 (16)	O1—C17—C18—C19	−178.06 (14)
C1—C6—C7—C12	−0.10 (18)	C16—C17—C18—C19	1.0 (2)
C12—C7—C8—C9	0.7 (2)	C17—C18—C19—C14	−0.2 (2)
C6—C7—C8—C9	−179.79 (16)	C15—C14—C19—C18	−0.7 (2)
C7—C8—C9—C10	−0.7 (3)	C13—C14—C19—C18	174.99 (14)
C8—C9—C10—C11	−0.3 (3)	C17—O1—C20—C21	−178.77 (13)
C9—C10—C11—C12	1.4 (3)	C12—C13—C23—C24	86.88 (17)
C10—C11—C12—C7	−1.4 (2)	C14—C13—C23—C24	−147.28 (15)
C10—C11—C12—C13	−178.96 (15)	C1—C13—C23—C24	−23.34 (19)
C8—C7—C12—C11	0.3 (2)	C12—C13—C23—C28	−86.21 (17)
C6—C7—C12—C11	−179.24 (14)	C14—C13—C23—C28	39.6 (2)
C8—C7—C12—C13	178.31 (14)	C1—C13—C23—C28	163.57 (15)
C6—C7—C12—C13	−1.28 (18)	C28—C23—C24—C25	1.5 (2)
C11—C12—C13—C14	−63.3 (2)	C13—C23—C24—C25	−171.88 (15)
C7—C12—C13—C14	118.92 (15)	C23—C24—C25—C26	−0.3 (2)
C11—C12—C13—C1	179.74 (16)	C29—O2—C26—C27	178.32 (14)
C7—C12—C13—C1	1.99 (16)	C29—O2—C26—C25	−2.0 (2)
C11—C12—C13—C23	62.0 (2)	C24—C25—C26—C27	−1.6 (2)
C7—C12—C13—C23	−115.75 (15)	C24—C25—C26—O2	178.69 (15)
C2—C1—C13—C12	177.43 (15)	O2—C26—C27—C28	−178.08 (15)
C6—C1—C13—C12	−2.06 (16)	C25—C26—C27—C28	2.2 (3)
C2—C1—C13—C14	57.5 (2)	C26—C27—C28—C23	−0.9 (3)
C6—C1—C13—C14	−121.99 (14)	C24—C23—C28—C27	−0.9 (2)
C2—C1—C13—C23	−68.3 (2)	C13—C23—C28—C27	172.36 (15)
C6—C1—C13—C23	112.26 (14)	C26—O2—C29—C30	−64.78 (19)

### Hydrogen-bond geometry (Å, °)

**Table:**

*D*—H···*A*	*D*—H	H···*A*	*D*···*A*	*D*—H···*A*
C29—H29A···O1^i^	0.99	2.46	3.358 (2)	151

Symmetry codes: (i) −*x*+2, *y*+1/2, −*z*+3/2.

## References

[bb1] Barbour, L. J. (2001). *J. Supramol. Chem.* **1**, 189–191.

[bb2] Bruker (2009). *SAINT* and *SMART* Bruker AXS Inc., Madison, Wisconsin, USA.

[bb3] Hamciuc, C., Hamciuc, E., Ipate, A. M., Cristea, M. & Okrasa, L. (2009). *J. Appl. Polym. Sci.* **113**, 383–391.

[bb4] Shah, K., Yousuf, S., Raza Shah, M. & Ng, S. W. (2010). *Acta Cryst.* E**66**, o1705. 10.1107/S1600536810022579 PMC3006837 21587925

[bb5] Sheldrick, G. M. (2008). *Acta Cryst.* A**64**, 112–122. 10.1107/S0108767307043930 18156677

[bb6] Westrip, S. P. (2010). *J. Appl. Cryst.* **43**, 920–925.

